# Managing Occupational Health Issues through Coaching, Emerging Perspectives from Emergency and Intensive Care Nurses: A Mixed-Method Study

**DOI:** 10.3390/nursrep13030094

**Published:** 2023-08-14

**Authors:** Rabia Chahbounia, Abdellah Gantare

**Affiliations:** Laboratory of Health Sciences and Technologies, Higher Institute of Health Sciences, Hassan First University of Settat, Settat 26000, Morocco; abdellah.gantare@uhp.ac.ma

**Keywords:** occupational health issues, stress, burnout, coaching needs, grounded theory approach, nursing staff

## Abstract

(1) Background: Emergency and intensive care nurses are among the health professionals most exposed to occupational health issues such as stress and burnout, etc. Coaching has been considered a useful preventative strategy to provide better support for professionals. This study has two objectives: the first objective is to identify the coaching needs of emergency and intensive care nurses, and the second is to propose a coaching model that addresses the needs and helps manage occupational health issues. (2) Methods: this study followed a mixed-method design, and it included thirty nurses working in the emergency and intensive care unit from two public hospitals in Morocco. The study entailed semi-structured interviews transcribed verbatim until data saturation, guided by the grounded theory approach in order to explore the coaching requirements of emergency and intensive care nurses, and the measurement of the three dimensions of burnout with the Maslach Burnout Inventory (MBI). (3) Results: the results reveal three main conceptualizing explanatory categories of the nurses’ coaching requirements: Steps of a coaching action; topics for a coaching action related to occupational health issues such as stress and burnout (it is shown that the prevalence of burnout in our sample is 66.7%); barriers to a coaching action. (4) Conclusions: by investigating the coaching requirements of the nursing staff, a transtheoretical coaching model with a theoretical and ethical basis was suggested in this regard for their occupational health issues management.

## 1. Introduction

The nursing profession is prone to stress. This is due to the intense nature of the work. The massive demands come from both internal and external sources. These demands add to the complexity of the job [1].

It is commonly known that nurses work under difficult conditions, including time constraints, a fast-paced environment, long hours, etc. [1].

From this perspective, the emergency department is recognized as highly stressful. The intensive care unit is also considered a highly stressful area of nursing. This is due to the constantly changing, highly systemized, and demanding environment [2].

Research has identified several causes of stress among emergency departments and intensive care unit nurses; these include interpersonal conflicts between colleagues, constant demands from superiors, highly demanding routines, a lack of human resources, a heavy workload, witnessing, the death and suffering of patients, falls, medication errors [2], etc. As a consequence, nurses are exposed to stressful and traumatic events. These exposures lead to a variety of health occupational issues. Such issues manifest as physical and mental health disorders (e.g., heart disease, pains, stomach complaints, muscle cramps and spasms, sleep disturbances, irritability, loss of concentration, anxiety, depression, compassion fatigue, burnout, etc.) [3,4].

All these factors can contribute to the inability to provide high-quality nursing care, job dissatisfaction, absenteeism, and, ultimately, increased nursing staff turnover rates [3].

As a matter of fact, in Morocco, emergency and intensive care nurses are the most exposed to occupational health problems compared to other categories of healthcare providers [5]. As a result, they become vulnerable and subject to psychological suffering such as burnout.

Following a study carried out by Nouhaila Syrine El Hijazi among care workers in emergency departments in the Souss-Massa area in Morocco [6] (p. 5), it was found that 15.1% had high levels of stress, while 81.1% had moderate levels of stress [6] (p. 26); 39.6% had a high emotional exhaustion score, 50.9% had a high depersonalization score, and 69.8% had a low personal accomplishment score [6] (p. 35).

Furthermore, Chakhtoura Khalid’s study reveals that emergency nurses are more stressed than other categories of caregivers [7].

Therefore, nursing tasks are complex and dynamic. For this reason, nurses require a supportive process, which helps them continually strive for a balance of personal expectations, professional concerns, and nursing realities [8].

On the one hand, among the most common interventions used are psychological skills training, such as coping/resilience training, cognitive behavioral therapy, mindfulness-based and meditation training, energy therapy (e.g., healing touch), yoga, other physical activity training, communication training, and so forth [9].

On the other hand, coaching enhances nurses’ competencies, individually and collectively. It improves communication and helps avoid psychological concerns by managing work-related stress. Coaching also assists in developing stress management skills [10].

The emphasis is on individual needs, strengths, and shortcomings through dialogue and reflection in a space of confidentiality and trust, and it improves productivity, knowledge, quality of life, the use of available skills and resources, the ability to adapt to circumstances and changes, leadership development, ensuring team cohesion, managing professional development, etc. [10].

Coaching is seen as a preventive strategy. It aims to offer better support to professionals. It also helps to limit the harmful consequences of occupational issues [11].

Although there is a great diversity of coaching theories, practices, and strategies, it is difficult to prejudge the effectiveness of one compared to another [12].

For this reason, this study intended to explore the coaching needs of emergency and intensive care nurses, in order to propose a coaching model for helping them better manage and circumvent their occupational health issues.

## 2. Materials and Methods

### 2.1. Design

This study used an exploratory mixed-method design. It aimed to investigate the coaching requirements of emergency and intensive care nurses. The aim of this study was to propose a coaching model for managing their occupational health issues.

Mixed-methods study is a research method that combines and integrates qualitative and quantitative research methods in a single research study, in order to fully comprehend a phenomenon and respond to various kinds of research questions [13].

In an exploratory mixed-method research design, we gather and analyze qualitative data before following up on our findings with a quantitative phase. This design for mixed qualitative and quantitative research tries to investigate a phenomenon before identifying which variables we need to measure quantitatively, leading to the integration or connection of data from the two distinct corpora to further explore, develop, and evaluate the qualitative analysis [13].

Qualitative research is based on the inquiry process that explores a social or human problem. The study is based on observations and interpretations of people’s perceptions of various events. We construct a complex, holistic picture, analyze words, report details of informants, and conduct the study in a natural setting [14].

According to this perspective, the qualitative research method uses the grounded theory approach. This approach was developed by sociologists Barney Glaser and Anselm Strauss. They defined grounded theory as theory derived from data systematically gathered and analyzed during the research process (Strauss & Corbin 1990 [15]). The aim is to construct a theory that is grounded in the data [14].

Quantitative research is regarded as the organized inquiry about phenomena through the collection of numerical data and execution of statistical, mathematical, or computational techniques. It incorporates statistical analysis with other techniques such as inferential statistics, hypothesis testing, experimental and quasi-experimental design randomization, structured protocols, questionnaires, etc., with a limited range of prearranged answers [16].

In line with the mixed-methods design, each component had its own research question. The qualitative component question was the following: What are the coaching requirements of emergency and intensive care nurses regarding their occupational health issues management?

The qualitative research method approach of grounded theory was used to explore the coaching requirements of nurses working in highly stressful departments like emergency and intensive care. Our goal was to identify the coaching actions needed to address their specific challenges and propose a coaching model that can effectively alleviate and manage their occupational health issues.

The quantitative component was complementary to the qualitative study, and it explored the 3 dimensions of burnout (BO), the frequency of BO, and the intensity of BO, as burnout was found to be one of the most relevant and recurrent occupational health issues among the participants retained from the qualitative research, as determined by the recurrence of stress and depressive symptoms among participants.

For this reason, participants were invited to fill out the Maslach Burnout Inventory (MBI) in order to evaluate the psychological impact of their work experiences while researching the effects of stress reported by participants.

Participants meeting the inclusion criteria were recruited through purposeful sampling, a technique widely used in qualitative research to identify and select information-rich cases efficiently with limited resources.

For this study, 30 Moroccan nurses working in the most stressful departments (emergency and intensive care units) were recruited. These nurses encounter the most recurrent occupational health issues, such as stress and burnout. The recruitment process involved contacting supervisors and head nurses to find individuals who were collaborative, available, willing to participate, and able to communicate experiences and opinions in an articulate, expressive, and reflective manner.

Among them, 21 were working in emergency departments and 9 in intensive care units. A total of 30 nurses met the inclusion criteria (see Table 1).

### 2.2. Data Collection

Data were collected by two researchers from 1 October 2021 to 30 November 2021. The first researcher was a coach in human development, a doctoral student, and a nurse practicing in an emergency department within a public hospital in Morocco Country; the second holds a Ph.D. in Communication Terminology and Didactic Engineering, was head of the research unit Nursing and Midwifery Sciences, and was a trainer and researcher in qualitative research at a higher institute of health sciences in Morocco.

#### 2.2.1. Qualitative Study

The semi-structured interviews were carried out face-to-face with each participant individually. They contained self-developed open-ended questions (17 items) based on a study of the relevant literature and further developed by discussion within a group of experts (in addition to the authors, a general nurse/coach in human development and a neuro-linguistic-programming–soft-skills hypnotherapist; the first author was a general practitioner, emergency nurse, and human development coach, and the second author holds a Ph.D. in Communication Terminology and Didactic Engineering, and was the head of the Nursing and Midwifery Sciences Research Unit). Data included additional observations and field notes about personal, organizational, and relational problems.

Each interview lasted approximately 25 to 30 min, and was conducted in a secure and calm place in both hospitals.

The interview guide consisted of five parts: (a) socio-demographic questions (5 items); (b) problems inherent to the activity of caregivers (4 items); (c) the accompaniment of nurses in demanding departments (emergency and intensive care units) (2 items); (d) methods of supporting nurses (2 items); (e) the feasibility of implementing a coaching model dedicated to nurses working in emergency and intensive care units (4 items) (Interview S1).

#### 2.2.2. Quantitative Study

The thirty participants were invited to fill out the Maslach Burnout Inventory (MBI) to assess psychological harm at work while researching the effects of chronic stress reported by participants.

The data collection was ensured by the first author.

The Maslach Burnout Inventory (MBI) is used with 22 items to evaluate the three components of BO syndrome: emotional exhaustion (9 items), depersonalization (5 items), and personal accomplishment (8 items). In our study, the MBI was used in its French version because our sample was fluent in French. Responses for each dimension are given on a 7-point frequency scale, from “never” to “every day” [17] (p. 193).

Each dimension’s score indicates the level of severity, classified as low, moderate, or high. Emotional exhaustion is classified as low when the score is below 17, moderate when it is between 18 and 29, and high when it is above 30. Depersonalization is considered low when the total score is less than 5, moderate when it is between 6 and 11, and high for a total exceeding 12. Personal accomplishment is deemed low if the score is above 40, moderate for scores between 34 and 39, and high when scores are below 33 [18].

The presence of a high score in emotional exhaustion or depersonalization, or a low score in personal accomplishment, is sufficient to define burnout (BO). If any one of the three dimensions is pathological, BO is considered “low”. If two of the three dimensions are pathological, it is categorized as “medium,” and if all three dimensions are pathological, it is termed “high” [18].

In this study, individuals were categorized as professionally exhausted if they displayed at least one pathological dimension. Burnout (BO) was classified as high or severe when both emotional exhaustion and depersonalization were elevated, combined with low levels of personal accomplishment.

### 2.3. Scientific Rigor

All the interviews conducted ensured consistency in data collection and interpretation.

In the present study, four supporting processes of trustworthiness were applied, namely credibility, dependability, confirmability, and transferability [19].

In this study, “credibility” was achieved by accurately and truthfully depicting participants’ lived experiences. To ensure this, prolonged engagement and persistent observation were employed to understand the context of the phenomenon and minimize data distortions. One author spent over two months with intensive care nurses, performing preliminary research. This allowed for a better understanding of their occupational health issues and critical needs. Discussions about the research aim and its usefulness were held with the second researcher. To maintain the validity of the data and interpretations, both researchers (the first researcher trained in qualitative research and the second researcher experienced in qualitative research) cross-checked the information across each category of participants.

“Transferability” was enhanced by using purposive sampling through the selection of information-rich cases of emergency and intensive care nurses who were more exposed to occupational health issues, after having contacted supervisors and head nurses in order to identify and select individuals that could be collaborative, available, willing to participate, and able to communicate their experiences about occupational health issues, coaching needs, and opinions in an articulate, expressive, and reflective manner.

In this study, recruitment of participants and data collection continued until the data were saturated, completed, and replicated by interviewing additional participants in order to increase the scope and appropriateness of the data. We immersed ourselves in the phenomenon to know, describe, and understand it fully. Special care was given to the collection, identification, and analysis of all data pertinent to the study. The interviews were transcribed verbatim and analyzed utilizing Corbin and Strauss’s approach to grounded theory [15].

“Dependability” was achieved by the two researchers. To be able to analyze and validate our findings related to the themes of the research question, a doctoral-prepared nursing colleague was asked to review some of the transcribed materials. Any new data descriptors illuminated by our colleague were acknowledged and considered. The results were then compared with our own thematic analysis from the entirety of the reviewed participant’s transcribed data. If the data findings identified by the colleague did not appear in our own thematic analysis, it was agreed by both researchers not to use the said theme. Our goal was that both researchers should agree on the findings related to the data findings and meanings within the transcribed material.

“Confirmability” was achieved by the first researcher maintaining a reflexive journal during the research process; she retired to a private room for the process of collecting and adding new codes, the results of which were also verified by the second researcher and other colleagues in the nursing research field. Furthermore, the process of the research was validated by an audit trail of a Research Ethics Committee.

### 2.4. Ethical Approval

This study adheres to the principles outlined in the Declaration of Helsinki [20]. Verbal informed consent was obtained from all the nurses to participate in the study and for using the data. Ethical approval was obtained from the Institutional Review Board (IRB) of the Moroccan Association for Research and Ethics, Research Ethics Committee (IRB00012973 Moroccan Association for Research and Ethics IRB #1) (No.11/REC/21) on 27 September 2021.

### 2.5. Data Analysis

#### 2.5.1. Qualitative Data Analysis

For the qualitative data analysis, the interviews were transcribed verbatim and analyzed utilizing Corbin and Strauss’s approach (1990) to grounded theory [15]. Data collection was realized in parallel to analysis and continued until data saturation was reached. Transcript analysis was conducted utilizing the Atlas-ti program, version 9 and relied on three coding methods: open coding, axial coding, and selective guided coding. Throughout the open coding, core characteristics of the interview data were captured. Based on the initial codes, core categories were developed using axial coding. As a final step, the relationships within the main categories were identified for the theorization of the coaching requirements of the study sample through selective coding. During the coding process, a constant comparison was also undertaken between data, codes, and categories.

Conceptual thinking and theory building are interrelated with the qualitative-research-method-approach-grounded theory. The researchers tried to highlight and explore the nurses’ perceptions regarding their coaching needs in the context of health occupational issues management, and took a snapshot of the people’s perceptions regarding their work environment (public hospitals) [14].

In this study, through the qualitative-research-method-approach-grounded theory, we sought to explore the coaching requirements of nurses working in the most stressful departments (emergency and intensive care departments) to which a coaching action must respond, in order to suggest a coaching model that could be suitable for their coaching requirements and helpful in alleviating and managing their occupational health issues.

We used the qualitative-research-method-approach-grounded theory to explore the coaching requirements of nurses working in highly stressful departments like emergency and intensive care. Our goal was to identify the coaching actions needed to address their specific challenges and propose a coaching model that can effectively alleviate and manage their occupational health issues.

For this reason, we pointed out the perceptions and coaching requirements of nurses after examining 30 interviews in depth, through Atlas-ti software version 9. The interviews were transcribed verbatim and analyzed utilizing Corbin and Strauss’s approach to grounded theory [15].

Data collection was realized in parallel to analysis and continued until data saturation was reached. We found that code saturation was reached at seventeen, at which point no new codes occur in the data. However, more data were collected to explore and confirm existing findings. The repeated collection of similar data indicates that we have saturated all that we can learn about a topic, and that additional data do not lead to any new emergent themes.

Transcript analysis was conducted utilizing the Atlas-ti program, version 9 and relied on three coding methods: open coding, axial coding, and selective guided coding. Throughout the open coding, core characteristics of the interview data were captured. Based on the initial codes, core categories were developed using axial coding. As a final step, the relationships of the main categories were identified for the theorization of the coaching requirements of the study sample through selective coding. During the coding process, a constant comparison was also undertaken between data, codes, and categories.

#### 2.5.2. Quantitative Data Analysis

Quantitative data from our sample related to three components of the Maslach Burnout Inventory (MBI) that entailed burnout syndrome, namely emotional exhaustion, depersonalization, and personal accomplishment, were entered and processed using the SPSS version 20 software.

## 3. Results

### 3.1. Socio-Demographic Characteristics

Our study included 30 nurses working in two departments (emergency and intensive care units) of two public hospitals in the Settat area of Morocco. The socio-demographic characteristics of our sample are summarized in Table 2.

### 3.2. Qualitative Findings

#### Conceptualizing Categories of the Coaching Requirements of Emergency and Intensive Care Nurses

A model was developed to explain nurses’ coaching requirements. These requirements were based on their perceptions and observed or expressed needs. Data were presented using Corbin and Strauss’s (1990) framework [15]. This framework helped develop conceptual categories and subcategories explaining nurses’ expressed coaching requirements.

The thorough transcript analysis relied on three coding methods: open coding, axial coding, and selective coding.

Open coding was used line-by-line throughout the interview transcripts; initial coding was needed to accurately preserve participants’ words, actions, and processes. Then, axial coding was used to organize the codes developed in open coding, and to draw connections between codes to determine how our codes can be grouped into categories, and selective coding was used to identify core categories, patterns, and relationships that emerged through informal clustering and mind mapping. Finally, theoretical coding, with a predominant central category, core categories, and subcategories, was used to find constructs and connections and explain relationships to generate conceptual frameworks that entailed a central category and core categories, which explain the coaching requirements for the implementation of a coaching action based on nurses’ expectations.

According to this perspective, there is a predominant and central category: “Coaching needs”. This category refers to the requirements for a coaching action based on nurses’ expectations and needs.

**Figure 1:**
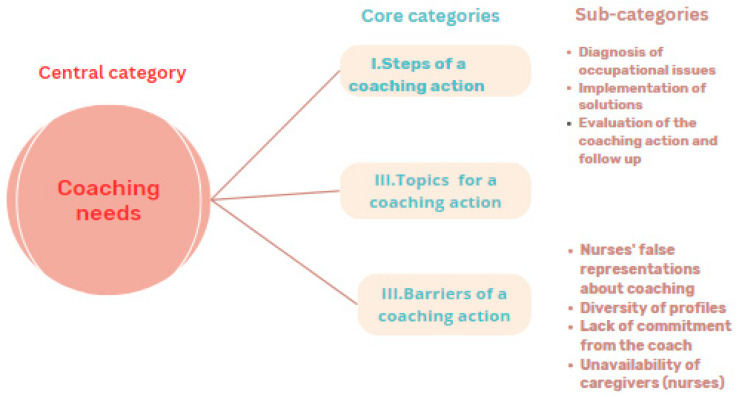
Nurses coaching needs/requirements.

In this regard, according to nurses, the coaching action must meet the following coaching requirements, explained in three core categories (Figure 1):

(I) Steps of a coaching action;
(II) Topics for a coaching action;
(III) Barriers to a coaching action: challenges to be dealt with for implementing a coaching action.

(I) Steps of a coaching action: this category explains the essential stages of a coaching action according to participants’ requirements, and involves three subcategories:
  ❖ Diagnosis of occupational issues;
  ❖ Implementation of solutions/dealing with occupational health issues;
  ❖ Evaluation of the coaching action and follow-up.

Diagnosis of occupational issues

This subcategory underlines the problems inherent to the activity of nurses; it corresponds to three categories of occupational issues: personal, organizational, and relational problems.

- Personal problems are associated with:
  - Emotional and psychological disorders
    Based on the experiences of the participants, some of them find themselves in a situation of stress, anxiety, depression, and burnout.
  - The lack of motivation and self-efficacy
    In this regard, some of them have a lack of motivation and self-efficacy due to the lack of recognition from authority figures, patients, and family; this was proposed by some participants.
- Organizational factors:
  The organizational factors are related to:
  - Communication problems with colleagues: The nurses revealed mainly inter-professional communication problems with colleagues who cause conflicts with colleagues, and poor inter-team coordination.
  - Deployable working conditions: Workload, lack of human resources and materials, and lack of hygiene were cited
  - Poor management: Problems with the distribution of tasks and a gap between pedagogical achievements and reality were described.
- Relational factors:
  Relational factors are mainly related to patients and their families, including prejudice and lack of respect.
  - Prejudice: Due to prejudices, incorrect ideas emerged—a lack of trust in the caregiver and illiteracy were cited.
  - Lack of respect towards the care provider: This causes communication problems, aggression towards caregivers by patients and families, and psychological harassment.

❖ Implementation of solutions/dealing with occupational health issues;
  This subcategory is related to:
  - The action of coaching by a nursing coach and undertaking weekly sessions (this defines the frequency of the coaching action that is part of the contract);
  - The resolution of the issues;
❖ Evaluation of the coaching action and follow-up:
  Some nurses declared that they want to have a follow-up after the coaching action.

(II) Topics for a coaching action: this category determined the main themes of the nurses’ suggestions to be addressed during a coaching action related to occupational health issues (stress, conflict at work, low self-esteem).

It has been found that this category is associated with the category of “steps of a coaching action”.

Some nurses find that coaching could help them circumvent the following issues and risk factors that could lead to burnout: stress, conflict and communication problems at work, low self-esteem, etc. through their integration in the different steps of the coaching action process.

(III) Barriers to the coaching action: this category focuses on the challenges to be dealt with in order to implement a coaching action, which make the process of a coaching action difficult.

Four subcategories were extracted from the category of barriers to a coaching action according to the nurses interviewed that might prevent them from meeting their coaching needs:

❖ Nurses’ false representations about coaching: due to misconceptions about coaching and a lack of awareness of the usefulness of coaching for some nurses.
❖ Diversity of profiles: due to the diversity of personalities and problems encountered by each person.
❖ Lack of commitment from the coach: some of the interviewees revealed that the non-commitment of the coach or the lack of a health coach could obstruct the action of the coaching.
❖ Unavailability of caregivers (nurses): due to the inconvenient working hours, the workload, the indifference of the staff, and their resistance to change.

### 3.3. Quantitative Findings

#### Occupational Health Issues: Stress–Burnout–Depression

All the participants find work stressful (30 participants (100%)), and 16 of them (a rate of 53.3%) are unable to protect themselves against stress. It is confirmed that the prevalence of burnout is 66.7%.

Among our sample, 20 nurses were in a state of moderate or high burnout (16 of them working in the emergency department and 4 in the intensive care unit had burnout); 6 of them were experiencing high or severe burnout, and 14 nurses were in a state of moderate or average burnout. The intensity of the three dimensions of burnout (emotional exhaustion, depersonalization, and personal accomplishment) is shown in Table 3 below:

Furthermore, the majority of participants had the most recurrent symptoms of depression shown by Denton et al. [21], and reported by the participants according to their number of occurrences: sadness (n = 15), loss of interest (n = 10), low self-esteem (n = 12), feelings of guilt (n = 9), sleep or appetite problems (n = 20), fatigue (n = 28), poor concentration (n = 16).

## 4. Discussion

Based on our sample of nurses’ coaching requirements, it was deduced that a coaching action could meet the following requirements identified in three core categories:

* Steps/components of a coaching action that were described in three subcategories: diagnosis of personal, organizational, and relational occupational issues; implementation of solutions/dealing with occupational health issues; evaluation of the coaching action and follow-up.
* Topics for a coaching action to be addressed during a coaching action related to occupational health issues (stress, communication problems, low self-esteem, etc.).
* Challenges to be dealt with for implementing a coaching action: nurses’ false representations about coaching, the diversity of profiles, nurses’ fear of the coach’s non-commitment, and the unavailability of caregivers.

According to this perspective, a transtheoretical coaching model, Pro-action, Interaction, and Retro-action (PIR), was suggested. The goal is to carry out effective coaching actions that meet nurses’ coaching requirements and help them manage various occupational issues. This model has proven to be effective in addressing stress among nurses in the emergency department [22].

It has been found that the aforementioned transtheoretical (PIR) model, which was elaborated from the most scientific coaching models that have theoretical and ethical bases (Dojo model, PAIR model, ABC model, GROW model, PRACTICE model, and Peer Coaching Model) [22], could be appropriate for the coaching requirements of nurses. Suggestions and expectations explained in the mentioned categories and subcategories can be summarized as follows: to be organized, with steps to follow, to have the objective of addressing the topics and the issues related to the caregiver’s activity, and also to deal with the barriers that may prevent the implementation of the coaching action.

In this regard, the transtheoretical (PIR) coaching model has three fundamental steps (Pro-action, Interaction, and Retro-action), where each fundamental step includes different sub-steps.

Beginning with the first fundamental step, pro-action includes three phases: “Welcoming and building confidence”, “The problem identification phase”, “Negotiation and establishment of the contract”.

“Welcoming and building confidence”: This phase allows coaches to gain the trust of the coachees [23], and to deal with the barriers to a coaching action in terms of nurses’ false representations about coaching, the diversity of profiles, and the nurses’ fear of the lack of coach’s commitment.

“The problem identification phase”: This phase is used to meet the nurses’ expectations from coaching in terms of diagnosis [24], in order to identify personal, organizational, and relational occupational issues.

“Negotiation and establishment of the contract”: This phase comes to complete the mentioned phases in order to specify the frequency, the duration, and the different topics of occupational issues to be handled in a coaching action [23]. This ensures adaptation to the coachees’ different profiles, and it outlines the commitments of both the coachees and the coach during the coaching action in order to deal with nurses’ fears of the coach’s non-commitment as a barrier to a coaching action.

The fundamental interaction step entails four subcategories: increasing self-efficacy, neutralizing obstacles, options, and implementation of the chosen solutions.

“Increasing self-efficacy phase”: This subcategory refers to a specific concept of Bandura’s learning theory [25] (p. 70); it is important in order to reinforce nurses’ motivation and increase their self-efficacy.

“Neutralizing obstacles”: In this regard, different coaching strategies have been involved, e.g., positive personal forces, including things that give us pleasure and meaning [26] (p. 9). This subcategory emanates from Peterson’s psychometric model of positive psychology [26] (p. 12); it includes concepts such as teamwork, social intelligence leadership, self-control, optimism, spirituality, [26] (p. 12), Neuro-Linguistic Programming exercises, and so forth.

“Option”: This step allows the suggestion of solutions to the diagnosed problems (Palmer 2008 [24]).

“The implementation of the chosen solutions phase”: This step allows management of the identified problems [24].

The last fundamental step, retro-action, includes two phases, “Evaluation of the selected solutions” and “Ensuring the empowerment of the coachees”.

“Evaluation of the selected solutions”: This step aims to assess the chosen solutions [24].

“Ensuring the empowerment of the coachees”: This step aims to reinforce their autonomy [27] (p. 256).

The transtheoretical PIR coaching model combines the different coaching models through the integration of their different steps, and is appropriate for the nurses’ coaching needs, suggestions, and expectations regarding coping with different occupational issues. In this regard, a pilot study conducted by Rabia Chahbounia and Abdellah Gantare has shown the effectiveness of this model in dealing with the stress of emergency nurses as an occupational issue. They applied a coaching intervention and used a multiple-case replication in order to help them in managing their stress [22]. From this perspective, the outcomes demonstrate encouraging improvements in the post-questionnaire results following the implementation of a coaching intervention. The questionnaire was designed to evaluate nurses’ knowledge and skill levels regarding stress effects and management. The mean pre- and post-test scores showed a significant difference (*p* = 0.016). After participating in the coaching experience, nurses’ average scores increased by 2.86 points, rising from 3.71 in the pre-test to 6.57 in the post-test [22]. Thus, it has been shown that a coaching intervention using a transtheoretical coaching model could potentially be an efficient strategy for managing nurses’ occupational health issues. It takes also into consideration the orientation of the coachees towards psychologists and psychiatrists, who can better help them deal with their critical situations, especially if the coachee’s approach to these categories of caregivers continues outside the boundaries of coaching [27] (p. 256).

In addition to the research, conducted by Rabia Chahbounia and Abdellah Gantare in a unique center, on evaluating the efficacy of the coaching intervention on nurses’ stress management, guided by the transtheoretical model [22], the effectiveness of this transtheoretical coaching model needs to be evaluated for its ability to aid in coping with various occupational issues (stress, low self-esteem, communication problems, etc.). Hence, there is a need to carry out further research for the implementation of this model in multicenter studies.

## 5. Conclusions

Our research has identified three main conceptual explanatory categories of the coaching needs of nurses working in high-stress environments, which are as follows: (1) Steps of a coaching action. (2) Topics for a coaching action related to occupational health issues, such as stress and burnout, for which nurses require coaching. (3) Barriers to a coaching action.

Based on the investigation of nursing professionals’ coaching needs, we propose a transtheoretical coaching model (PIR) with a solid theoretical and ethical foundation. This model aims to address their coaching needs and manage their occupational health issues.

To assess the effectiveness of this transtheoretical coaching model in coping with various occupational issues, multicenter studies need to be conducted to evaluate the impact of its implementation.

## Figures and Tables

**Table 1:** Inclusion and exclusion criteria for identifying participants.

Inclusion Criteria	Exclusion Criteria
Nurses working in public hospitals in the Settat region of Morocco.	Not being a nurse working in public hospitals in the Settat region of Morocco.
Working in the emergency or intensive care unit.	Nurses working in other departments outside emergency and intensive care units.
Being state-registered, with national public certification.	Not being state-registered.
Acceptance of the invitation to participate in the study.	Not having national public certification.

**Table 2:** Socio-demographic characteristics of study participants.

Variables	Categories	Number of Staff
Age group	25–35 years old	16
	36–59 years old	9
	Over than 60 years old	5
Gender	Female	10
	Male	20
Marital status	Single	11
	Married	16
	Widowers	2
	Divorced	1
Working department	Intensive care unit	9
	Emergency department	21
Working years of experience	<5 years	9
	5–15 years	16
	Over than 16 years	5

**Table 3:** The intensity of the three dimensions of burnout (emotional exhaustion, depersonalization, and professional accomplishment.

The Intensity of the Three Dimensions of Burnout	Number of People with Emotional Exhaustion	Number of People with Depersonalization	Number of People with Personal Accomplishment
Low	0	4	10
Moderate	8	6	8
High	22	20	12

## Data Availability

All relevant datasets in this study are described in the manuscript.

## Supplementary Materials

The following supporting information can be downloaded at: https://www.mdpi.com/article/10.3390/nursrep13030094/s1, Interview S1. Exploring Nurses Challenges and Feasibility of Health Coaching in High-Stress Healthcare Environments: An Interview Study in Emergency and Intensive Care Units.

## Public Involvement Statement

There was no public involvement in this research.

## Guidelines and Standards Statement

This manuscript was drafted against the reporting guidelines outlined in the Standards for Reporting Qualitative Research (SRQR) for qualitative method research.
