# Supplementary material for: Managing Occupational Health Issues through Coaching, Emerging Perspectives from Emergency and Intensive Care Nurses: A Mixed-Method Study
Source: Nurs Rep. 2023 Aug 14;13(3):1077–89. doi: 10.3390/nursrep13030094 (PMC10443326; doi:10.3390/nursrep13030094)
Supplement: Supplementary file 1 [file nursrep-13-00094-s001.zip › nursrep-2503753-supplementary.pdf]

Interview S1: Exploring Nurses Challenges and Feasibility of Health Coaching in High-Stress Healthcare Environments: An Interview Study in Emergency and Intensive Care Units.

Axis I. Socio-demographic questions:

1. gender: M ☐ F ☐

2. age: ....

3. Marital status: ....

4. In which department do you work? Emergency ☐ Intensive care unit ☐

5. How long have you worked in this department? : ……………………………………………..

Axis II. Problems inherent to the activity of caregivers :

6. What are the problems inherent in your activity as a caregiver on
Personal
level? : ……………………………………………………………………………………………
……

Organizational
level : ……………………………………………………………………………………………
……

Relational level, with the patient and his
family ? : ………………………………………………………………………. …... ………………
……….

7. Do you find the work stressful? Yes ☐ No ☐

If yes, what are the specific factors that cause you
stress ?: …………………………………………………………………………………………
………

8. Do you find yourself under chronic stress? Yes ☐ No ☐

9. Do you feel : Sadness ☐ loss of interest ☐ low self-esteem ☐

feelings of guilt ☐ sleep or appetite disorders ☐ feelings of fatigue ☐
lack of concentration ☐

Axis III: Accompaniment of nurses in demanding departments (emergency and intensive care unit)

10. What do you think about supporting nurses in the structure where you work ?

……………………………………………………………………………………………………

11. Why is there a lack of health coaching?: ………

Axis IV: The methods of supporting nurses

12. Do you protect yourself against stress, burnout and depression at your workplace?

Yes☐ No ☐ If yes, how ?: ....

13 .Can you relieve your professional stress after work hours ? :

……………………………………………………………………………………………………

Yes ☐ No ☐

If yes, how ? :………………………………………………………………………………

Axis V: The feasibility of implementing a coaching model dedicated to nurses working in emergency and intensive care unit:

14. To what extent can health coaching alleviate burnout?

……………………………………………………………………………………………………

15. What could hinder a coaching intervention ?

…………………………………………………………………………………………............

16. What are your expectations of the coaching model ?

……………………………………………………………………………………………………

17. Do you have any additional comments ?

……………………………………………………………………………………………………
